# Retinoid and thiazolidinedione therapies in melanoma: an analysis of differential response based on nuclear hormone receptor expression

**DOI:** 10.1186/1476-4598-8-16

**Published:** 2009-03-06

**Authors:** Joshua P Klopper, Vibha Sharma, Andrew Berenz, William R Hays, Michele Loi, Umarani Pugazhenthi, Sherif Said, Bryan R Haugen

**Affiliations:** 1 Department of Medicine, Division of Endocrinology, Metabolism and Diabetes, University of Colorado Denver, Aurora, CO, USA; 2 University of Colorado Cancer Center, University of Colorado Denver, Aurora, CO, USA; 3 Department of Pathology, University of Colorado Denver, Aurora, CO, USA

## Abstract

**Background:**

Metastatic melanoma has a high mortality rate and suboptimal therapeutic options. Molecular targeting may be beneficial using the rexinoid LGD1069, a retinoid × receptor selective agonist, and thiazolidinediones (TZD), PPARγ selective ligands, as novel treatments.

**Results:**

Mouse xenograft models with human melanoma cell lines [A375(DRO) or M14(5–16)] were treated for 4 weeks with daily vehicle, RXR agonist (rexinoid, LGD1069, 30 mg/kg/d), PPARγ agonist (TZD, rosiglitazone, 10 mg/kg/d) or combination. A375(DRO) tumor growth was significantly inhibited by either ligand alone and the combination had an additive effect. M14(5–16) tumors only responded to LGD1069 100 mg/kg/day. A375(DRO) sublines resistant to rexinoid, TZD and combination were generated and all three sublines had reduced PPARγ expression but preserved RXR expression. shRNA knockdown of PPARγ or RXRγ attenuated the rexinoid, TZD and combination ligand-mediated decreased proliferation in A375(DRO) cells. Rexinoid (LGD1069) and retinoid (TTNPB) treatment of M14(5–16) cells resulted in decreased proliferation that was additive with combination of both rexinoid and retinoid. shRNA knockdown of RXRγ resulted in a decreased response to either ligand.

**Conclusion:**

A375 (DRO) melanoma cell growth is inhibited by rexinoid and TZD treatment, and this response is dependent on RXR and PPARγ receptor expression. M14 (5–16) melanoma cell growth is inhibited by rexinoid and retinoid treatment, and this response is dependent on RXR expression. These findings may help guide molecular-based treatment strategies in melanoma and provide insight for mechanisms of resistance to nuclear receptor targeted therapies in certain cancers.

## Background

Melanoma represents a significant public health problem with a rising incidence over the last 3 decades[1]. More than 7700 patients will die annually of this disease, almost all with metastases [2]. Much of the research and current treatment for advanced stage malignant melanoma has utilized immunomodulating strategies including the use of interferon-α, other cytokines and vaccines[3].

Our group has long been interested in the study of nuclear hormone receptor targeted therapy for the treatment of poorly differentiated cancer with a primary focus on the retinoid receptors and peroxisome-proliferator activated receptor gamma (PPARγ) as novel therapeutic targets. Retinoid receptors can be divided into two broad categories of retinoic acid receptors (RAR) and retinoid × receptors (RXR), each with three separate isotypes encoded by 6 different genes (RAR α, β, γ and RXR α, β, γ)[4,5]. We have evaluated the utility of both RAR and RXR specific ligands (rexinoids) as well as PPARγ ligands to decrease cancer cell proliferation, increase apoptosis and inhibit tumor growth with *in vitro *and *in vivo *experiments [6-8].

There is data to suggest that nuclear hormone receptors may be important and relevant targets in melanoma. RXRs have been described as "auxiliary" receptors that enhance DNA binding of RAR and other nuclear hormone receptors, including PPARγ [9]. More recent studies, however, showed that selective activation of RXR could lead to transcriptional activation, apoptosis and redifferentiation of embryonal carcinoma cells, and that the effects of RAR and RXR selective ligands in combination were additive [10]. LGD1069, a rexinoid known as bexarotene, is approved for use in cutaneous T-cell lymphoma and has been studied as adjuvant therapy for non-small cell lung cancer [11,12].

PPARγ receptors have been demonstrated in primary human tissues as in a study by Mossner *et al*. that showed positive immunostaining for PPARγ in 14/14 nevi, 10/11 primary melanoma lesions and 6/8 melanoma metastases[13]. Placha and colleagues performed *in vitro *proliferation analyses on two PPARγ expressing melanoma cells: WM35, a primary melanoma lesion and A375. Using an older generation TZD, ciglitazone (5 μM), there was a significant decrease in cell proliferation at 48 hours [14].

Recently, we have reported that our earlier data was largely based upon cell lines that have been misidentified. Two of our rexinoid responsive cell lines were DRO90-1 and BHP 5–16, likely sub-lines of the melanoma cell lines A375 and M14 respectively [15]. Both cell lines express RXRγ, which is associated with response to rexinoid therapy *in vitro *for BHP 5–16 and both *in vitro *and *in vivo *in DRO90-1[7,8]. Additionally, RARβ expression is associated with treatment response using the RAR selective ligand TTNPB *in vitro *in BHP 5–16 only[6]. DRO90-1 is unique in that it also expresses PPARγ, which is activated by the thiazolidinedione (TZD) class of drugs (PPARγ agonists). We have observed that rexinoid treatment of DRO90-1 yields a greater decrease in proliferation as compared to BHP 5–16 (RXRγ+, PPARγ-) with a component of this effect due to a decrease in S phase and an increase in G2/M phase of the cell cycle[6]. Additionally, combination therapy with rexinoid and TZD of DRO90-1 *in vitro *yields a synergistic antiproliferative and pro-apoptotic response [7]. DRO90-1 also has a dose dependent decrease in tumor growth to rexinoid *in vivo *[8].

In this report, we expand upon our *in vivo *nude mouse model that analyzes the response of BHP 5–16 and DRO90-1 xenograft tumors to LGD1069 and rosiglitazone (ROSI), a TZD. Additionally, we examine the functional importance of RXRγ and PPARγ receptors in these cell lines by a unique model of resistance to rexinoid and TZD therapies in DRO90-1, as well as nuclear hormone receptor inhibition by short hairpin RNA (shRNA) directed at PPARγ (shPPARγ) in DRO90-1 and RXRγ (shRXRγ) in both DRO90-1 and BHP 5–16[16].

## Results

### In vivo A375(DRO) tumor growth is synergistically inhibited by combination treatment with rexinoid and TZD

We have previously shown that A375(DRO) xenograft tumor growth is inhibited by 30 and 100 mg/kg/day of LGD1069 in a dose dependent manner [8]. Additionally, a combination of low-dose RXR and PPARγ agonists significantly inhibited growth of A375(DRO) cells *in vitro *[7]. Therefore, nude mice harboring established A375(DRO) tumors (100–200 mm^3^) were treated with LGD1069 30 mg/kg/day (as previously described), ROSI 10 mg/kg/day or a combination of the two drugs.

**Figure 1:**
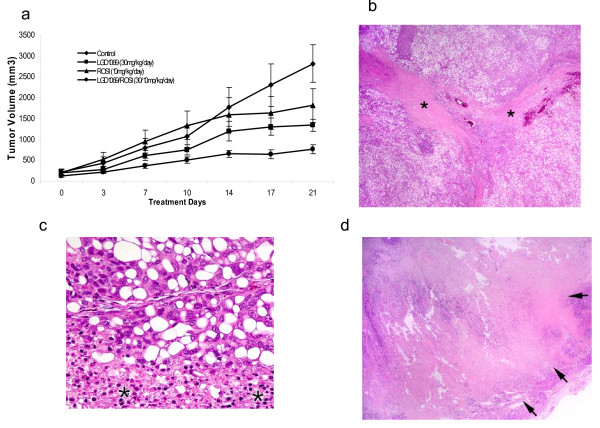
**1a: *In vivo *tumor response of A375(DRO) xenograft tumors**. 5 × 10^6 ^A375(DRO) cells were injected s.c. and tumors were allowed to grow for 3.5 wk after tumor establishment (~100 mm^3^). After estblishment, treatment conditions were (A) control, (B) 30 mg/kg/d LGD1069, and (C) 10 mg/kg/d ROSI and (D) combination of 30 mg/kg/day LGD1069 and 10 mg/kg/day ROSI. Tumors were measured twice per week. Points, mean tumor volume/group; bars, SEM. One Way ANOVA – Kruskal-Wallis test was used to assess the significance of the difference of tumor sizes at the end of the experiment (*P = 0.002). 1b: representative control tumor showing approximately 20% necrosis (*). Marked cellular vacuolization is seen in the non-necrotic tissue. Hematoxyline and Eosin staining 2× magnification. 1c: An enlarged picture from the control tumor showing the junction of the necrotic (*) and the viable, yet markedly vacuolated melanoma cells. Hematoxyline and Eosin staining. 40× magnification. 1d: A representative Rosi treated tumor with > 70% necrosis (edged by arrows) of the total tissue volume with a small rim of viable tumor. Hematoxyline and Eosin staining 2× magnification.

LGD1069 and ROSI treatments alone each had a modest effect on tumor growth (figs. 1a, 52% and 36% smaller than control respectively). Combination LGD1069 and ROSI inhibited tumor growth by 73% compared with controls. All tumor sizes at the end of the treatment were significantly smaller that untreated mice (p = 0.002, 1-way ANOVA Kruskal-Wallis method).

No metastases were observed grossly after an examination of lung and liver parenchyma in the control or treatment groups. Tumors in the ROSI or LGD1069 single therapy groups showed 57.5 ± 12.8% and 40 ± 12.2% necrosis respectively while the control tumors, though much larger, only showed 28.8 ± 14.6% necrosis. The control and LGD1069 treated tumors showed marked vacuolization in the viable tissue. Interestingly, the combination therapy group had the lowest amount of necrosis (21.3 + 15.3%) (p < 0.001 for all groups, 1-way ANOVA).

### An in vitro model of resistance to LGD1069 and TZD decreases A375(DRO) PPARγ expression

We have previously shown that 10 nM of LGD1069, PIO or the combination has no antiproliferative effects, but 100 nM and 1 μM of each drug alone and in combination inhibited cell growth by 90–98% compared to control [7]. Both PIO and ROSI have shown a similar inhibition of proliferation indicating a TZD class effect in this model (data not shown).

We generated drug resistant sub-lines of A375(DRO) by treating these cells in 10 nM of either ligand alone or in combination and increasing the concentration of drug as described in the methods until the cells had similar growth rates as cells grown in volume equivalent vehicle for the same period of time (~14 weeks). These cell lines were renamed based on the drug resistance generated (DMSO R – control cells, LGD1069 R – rexinoid resistance, TZD R – pioglitazone resistance and LG/TZD R – resistance to combination rexinoid and TZD).

**Figure 2:**
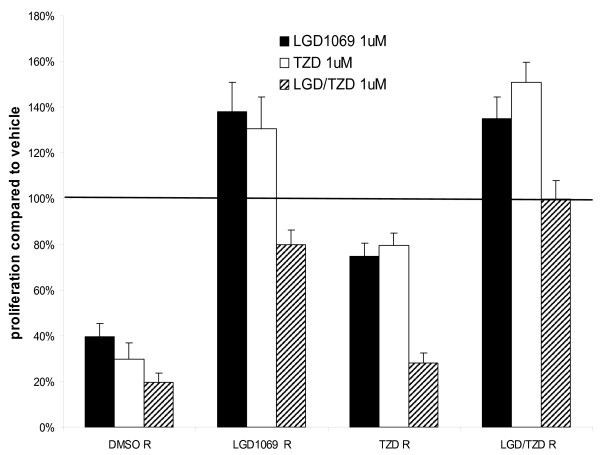
**Proliferation of resistant A375(DRO) cells: "resistant" cells were grown in 2% fetal bovine serum RPMI in the presence of 1 μmol/L of LGD1069, TZD or the combination for 9 days**. Cell growth was analyzed using a nonradioactive cell proliferation assay. Proliferation was compared to that of cells grown in volume equivalent vehicle (DMSO – represented by the line) and resistant sublines were compared to the DMSO R control to assess the attenuation of response to receptor specific ligands. Proliferation was statistically significantly attenuated compared to the control DMSO R in all resistant cell lines and condition save for combination therapy in TZD R (p < 0.03). Columns, mean; bars, SEM.

To confirm drug resistance, we performed proliferation assays with 1 μM of each ligand alone or in combination (10 × higher drug concentration than the final growing conditions of 100 nM). We first confirmed that the control A375(DRO) cells that were grown in volume equivalent vehicle had a similar response to early passage A375(DRO) cells exposed to volume equivalent vehicle (DMSO) (data not shown). Figure 2 shows that LGD1069 R cells were resistant to growth inhibition by 1 μM LGD1069 as expected, but surprisingly also lost the growth inhibitory effect of TZD, despite no exposure to TZD. These LGD1069 R cells also had significant attenuation of growth inhibition by LGD/TZD combination. TZD R cells attenuated growth inhibition by LGD and TZD alone compared to DMSO R cells. LGD/TZD R cells were completely resistant to growth inhibition by all conditions as was expected. The attenuation of the treatment effect to all treatment conditions compared to the control cell line (DMSO R) was significant (p < 0.03) except for the TZD R subline treated with combination LGD/TZD (fig 2).

**Figure 3:**
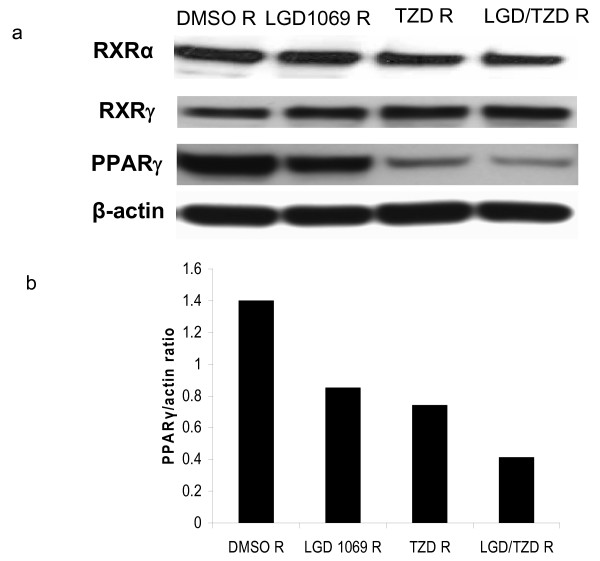
**Western blot of nuclear hormone receptors in A375(DRO) resistant cell lines**. 3a: 60 μg of nuclear protein extract from the resistant A375(DRO) sublines was size-separated on a 10% SDS-PAGE gel and transferred to nitrocellulose. The blot was blocked with 10% nonfat milk and incubated with RXRγ (MS-1343-P NeoMarkers) and RXRα (sc D-20) antibodies at a concentration of 1:500 and PPARγ (H-100) rabbit polyclonal ab (sc-7196, Santa Cruz Biotechnology, Santa Cruz, CA) at 1:500. Secondary antibodies were anti-rabbit IgG conjugated to horse-radish peroxidase at a 1:5000 dilution for RXRs and 1:1000 for PPARγ (GE Healthcare UK). β-Actin was measured as a loading control. 3b: PPARγ receptor to β-Actin ratio was calculated using an Alpha Innotech alpha imager.

We next examined nuclear protein levels of RXRα, RXRγ and PPARγ in each of the drug resistant sub-lines of A375(DRO) (fig 3a). Interestingly, RXRα and RXRγ protein levels were unaffected, but PPARγ was lower in all 3 sublines, with the LGD/TZD R cells having 71% less PPARγ receptor relative to DMSO R (fig 3b).

### shRNA for PPARγ and RXRγ in A375(DRO) attenuates both LGD1069 and ROSI treatment response to both ligands

In order to directly determine the roles and functional activity of PPARγ and RXRγ in treatment response to TZD and rexinoid in A375(DRO) cells, we performed shRNA knock-down of these receptors.

**Figure 4:**
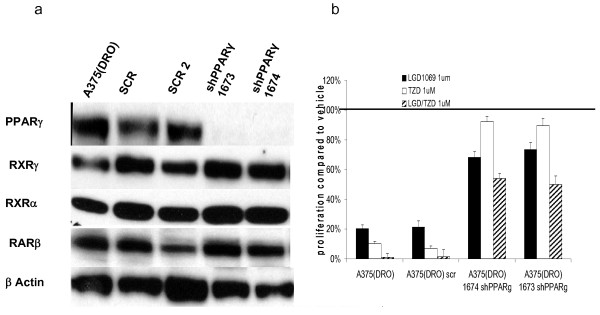
**Western blot of nuclear hormone receptors and proliferation in A375(DRO) cells with shPPARγ infection**. A. – 60 μg of nuclear protein extract from A375(DRO), the SCR shRNA infected control cell and two clones of shPPARγ infections. Proteins were size-separated on a 10% SDS-PAGE gel and transferred to nitrocellulose. The blot was blocked with 10% nonfat milk and incubated with RXRγ, RXRα and RARβ primary antibodies and then secondary antibody with anti-rabbit IgG conjugated to horse-radish peroxidase as previously described. β-actin was measured as a loading control. B. – A375(DRO), the SCR infected and two shPPARγ infected sublines were grown in 2% fetal bovine serum RPMI in the presence of 1 umol/L of LGD1069, TZD or the combination for 9 days. Cell growth was analyzed using a nonradioactive cell proliferation assay. Proliferation was compared to that of cells grown in volume equivalent vehicle (DMSO – represented by the line). Proliferation of the SCR infected A375(DRO) was compared to the native cell line to confirm a similar response and then the shPPARγ cell lines were compared to the SCR condition for an assessment of decreased proliferation. Proliferation was statistically significantly attenuated compared to the A375(DRO) SCR subline in all treatment conditions (p < 0.001). Columns, mean; bars, SEM.

Western blot analysis demonstrated loss of PPARγ protein in two distinct shPPARγ clones compared with scrambled shRNA control (SCR) or non-infected cells (fig 4a). We confirmed decreased mRNA by qRT-PCR to verify the loss of protein expression was occurring at the mRNA level (data not show). shPPARγ had no effect on RXRα, RXRγ or RARβ protein levels.

To assess the functional importance of PPARγ expression, we performed the proliferation assay as described (materials and methods) after 9 days of treatment using the SCR as a control for the lentiviral infection and two representative shPPARγ clones (1674 and 1673). A375(DRO) SCR control cells had a robust growth inhibitory response to ligand treatment that was similar to non-infected A375(DRO) cells. As expected, shPPARγ cells lacking PPARγ protein were no longer growth inhibited by TZD (fig 4b). Interestingly, growth inhibition by LGD1069 was significantly attenuated in these cells lacking PPARγ but with intact RXRα and RXRγ protein. These genetic experiments confirm our observations using the pharmacologic resistance model (fig 2 and 3). Growth inhibition by combination LGD/TZD was also attenuated in these cells lacking PPARγ.

**Figure 5:**
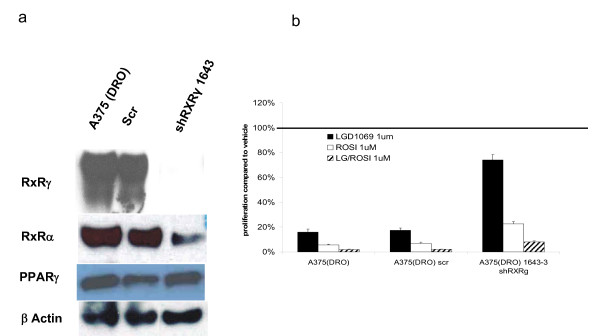
**Western blot of nuclear hormone receptors and proliferation in A375(DRO) cells with shRXRg infection**. A. – 60 μg of nuclear protein extract from A375(DRO), the SCR shRNA infected control cell and a clone of shRXRγ infected cells were size-separated on a 10% SDS-PAGE gel and transferred to nitrocellulose. The blot was blocked with 10% nonfat milk and incubated with RXRγ, RXRα and PPARγ primary antibodies and then secondary antibody with anti-rabbit IgG conjugated to horse-radish peroxidase as previously described. β-actin was measured as a loading control. B. – A375(DRO), the SCR infected and the shRXRγ infected sublines were grown in 2% fetal bovine serum RPMI in the presence of 1 umol/L of LGD1069, TZD or the combination for 9 days. Cell growth was analyzed using a nonradioactive cell proliferation assay. Proliferation was compared to that of cells grown in volume equivalent vehicle (DMSO – represented by the line). Proliferation of the SCR infected A375(DRO) was compared to the native cell line to confirm a similar response and then the shRXRγ infected cell line was compared to the SCR condition for an assessment of attenuation of decreased proliferation. Proliferation was statistically significantly attenuated compared to the A375(DRO) SCR subline in all treatment conditions (p < 0.05). Columns, mean; bars, SEM.

We next performed the converse experiment in A375(DRO) by using shRXRγ inhibition to knock down RXRγ protein. Infection with the shRXRγ clone 1643 resulted in loss of RXRγ protein expression in (fig. 5a). Figure 5b shows that knock-down of RXRγ resulted in significant attenuation, but not complete loss of inhibition of proliferation for each of the three ligand treatment conditions, indicating that RXRγ or some level of total RXR is necessary for the full suppressive effect of rexinoid, TZD and combination on A375(DRO) cell growth. shRXRγ infection appears to have partially decreased RXRα protein expression as well (likely via an indirect mechanism since shRXRγ are highly specific for RXRγ) which may contribute to the diminished LGD1069 effect.

### shRXRγ in M14(BHP 5–16) decreases the treatment effect of both LGD1069 and TTNPB monotherapy

We have previously demonstrated that *in vitro*, M14(5–16) does not respond to TZD and the antiproliferative effect to combination therapy with rexinoid and TZD is driven by the rexinoid effect[7]. These observations were confirmed in the *in vivo *xenograft where ROSI alone had no effect, nor did combination therapy with the lower dose of LGD1069 at 30 mg/kg/day combined with ROSI 10 mg/kg/day (data not shown). In the LGD1069 100 mg/kg/day treatment arm, there was a modest but significant effect on M14(5–16) tumor growth. Control tumors measured 1085 ± 68 mm^3 ^and treated tumors measured 866 ± 70 mm^3 ^(p = 0.04).

We have previously shown that the M14(5–16) responds to both RXR and RAR agonists with approximately 40–60% decreased proliferation compared to vehicle treated cells [6]. Based on our observations of the robust response of RXR:PPARγ heterodimer activation in A375(DRO), we evaluated if combination therapy with LGD1069 and TTNPB (RAR selective ligand) would result in a similar additive or synergistic antiproliferative response.

**Figure 6:**
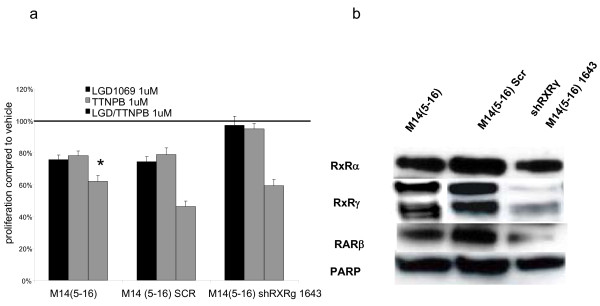
**Proliferation response of M14(5–16) with shRXRg infection and western blot of these cells**. A – M14(5–16), the SCR infected and the shRXRγ infected subline was grown in 2% fetal bovine serum RPMI in the presence of 1 μmol/L of LGD1069, TTNPB or the combination for 6 days. Cell growth was analyzed using a nonradioactive cell proliferation assay. Proliferation was compared to that of cells grown in volume equivalent vehicle (DMSO – represented by the line). Each treatment condition led to a significant decrease in proliferation compared to control (p < 0.001). The combination of LGD/TTNPB had a statistically significantly greater decrease in proliferation than each alone (*p = 0.02). Proliferation of the SCR infected M14(5–16) was compared to the native cell line to confirm a similar response and then the shRXRγ infected cell line was compared to the SCR condition for an assessment of attenuation of decreased proliferation. Proliferation was significantly attenuated compared to the M14(5–16) SCR subline monotherapy conditions (p ≤ 0.02). Columns, mean; bars, SEM. B – 60 μg of nuclear protein extract from M14(5–16), the SCR shRNA infected control cell and a clone of shRXRg infected cells were size-separated on a 10% SDS-PAGE gel and transferred to nitrocellulose. The blot was blocked with 10% nonfat milk and incubated with RXRγ and RXRα primary antibodies and then secondary antibody with anti-rabbit IgG conjugated to horse-radish peroxidase as previously described. RXRγ is represented as a doublet because of cross-reaction of the RXRγ_1 _and RXRγ_2 _isoforms. PARP was measured as a loading control.

We treated M14(5–16) with 1 μM LGD1069, TTNPB or the combination (500 nM of each) for 6 days. There was a modest but significant decrease in proliferation of 24%, 22% and 38% respectively compared to control conditions (p = 0.001). Additionally, the combination provided an additive response that was significant (16% reduction beyond either ligand alone compared to control, p = 0.02) (fig. 6a).

We then tested if shRXRγ would attenuate the antiproliferative response of LGD1069, TTNPB and the combination. We used the same shRXRγ clone (1643) that provided the greatest protein inhibition in the A375(DRO) cell line. Knock-down of RXRγ resulted in a significant attenuation of the treatment response for each treatment condition compared with SCR control cells (p ≤ 0.02) (fig. 6a).

Western blot analysis demonstrated significantly decreased RXRγ protein in M14(5–16) after infection with the 1643 shRXRγ clone. RXRα protein expression was maintained at a similar level to un-infected cells. However, RARβ expression was also decreased in the shRXRγ infected cells compared to the non-infected M14(5–16) and control SCR cells (fig. 6b).

## Discussion

In this report, we have demonstrated for the first time that the combination of a rexinoid and TZD effectively inhibits growth of differentiated melanoma expressing PPARγ and RXRγ using an *in vivo *model. Additionally, we have shown through a model of induced cell line resistance and shRNA mediated nuclear hormone receptor knockdown the requirement that both RXR and PPARγ are required for maximal effect of either ligand. Finally, we have demonstrated that a combination of a selective rexinoid and retinoid agonists has an additive effect on growth inhibition of a melanoma cell line expressing RARβ and RXRγ.

We have previously reported the efficacy of a combination of rexinoid and TZD to inhibit cell proliferation and promote apoptosis in A375(DRO)[7]. Additionally, we have demonstrated that LGD1069 inhibits A375(DRO) tumor growth in a nude mouse model[8]. We originally reported DRO as an anaplastic thyroid carcinoma, but we have recently shown by short tandem repeat (STR) analysis DRO matches the profile of A375, a poorly differentiated amelanotic melanoma that was established almost 20 years before the reported establishment of DRO [15].

Studies of the use of retinoids for melanoma therapy are limited. Niu and colleagues examined the *in vitro *effects of non-selective retinoids, an RXR selective agonist (methoprene acid) and the RAR selective agonist TTNPB on A375 cells [17]. They found that TTNPB and non-selective retinoids led to a greater antiproliferative and apoptotic response than the RXR selective agonist. This directly contradicts our data in that we have shown that RXR selective agonists leads to decreased cell growth and TTNPB does not in our A375(DRO) cells [6]. There are important differences in the two studies including different culture conditions, our use of a different RXR agonist and a no presentation of receptor expression by Niu *et al*. It is possible that genetic drift in culture has led to alterations in nuclear hormone receptor expression. Alternatively, the greatest response shown by Niu *et al*. was with 10 μM retinoid, which may be a concentration that exerts effects via non-receptor mediated mechanisms. Alternatively, in another melanoma cell line, S91, RXR selective ligands had a greater antiproliferative effect than RAR ligands[18].

The BHP 5–16 subline of the melanoma M14 responds to both RXR and RAR selective ligands (LGD1069 and TTNPB respectively) with decreased proliferation[6]. We assessed if the combination of these two ligands would lead to an additive or synergistic response via dual activation RXR and RAR receptors. Treatment with LGD1069 and TTNPB in combination at 1/2 the dose of each ligand used alone did lead to a modest and statistically significant decrease in proliferation, indicating at least an additive effect. Knockdown of RXRγ blunted the effect of both LGD1069 and TTNPB alone. The LGD1069 attenuation is easily explained by the decreased RXR expression. Despite the shRXRγ sequence being specific for RXRγ, we found lower protein levels of RARβ. The reason for this is unclear, but could possibly be explained by the requirement of functional RXR protein to be present for recruitment of RARβ from the cytoplasm to the nucleus for DNA binding (our proteins for western blot were enriched for nuclear extracts). Ikeda and colleagues demonstrated no detectable RARβ2 protein in S91 melanoma cells until treatment with an RXR selective agonist by dimethyl sulfate-based genomic footprinting[19]. Alternatively, intact RXR signaling may be required for RAR protein stability by an unknown mechanism.

There has been one clinical trial with rexinoid therapy and melanoma, a phase II study using bexarotene in 19 patients with metastatic melanoma [20]. This was a non-controlled study that evaluated the size and number of lesions by palpation or imaging after initiation with a maximum recommended dose of bexarotene. The best response seen was stable disease in one patient. Stable disease for malignant melanoma could be considered a significantly beneficial therapeutic outcome, although it was observed in only one patient and there was no untreated control group. Most importantly, there was no correlation of response (or lack of response) to RXR presence in either primary or metastatic lesions. Our data shows that RXRγ predicts response to LGD1069, though we cannot be certain if this is specific to RXRγ or total RXR protein levels.

A recent study using tissue microarrays gives an indication of the potential negative outcome in the above described trial[21]. Chakravarti *et al*. showed a significant decrease in RARβ, RARγ and RXRα expression in melanoma lesions compared to nevi. Additionally, of lesions with satellitosis, there was decreased RXRγ expression. The authors went on to show a decrease in overall survival in patients whose tumors had lower cytoplasmic staining for RARγ and RXRα. These data suggest that as melanoma becomes more aggressive and dedifferentiated, there is an associated loss of retinoid nuclear hormone receptor expression. Perhaps, earlier adjuvant therapy with retinoids in high risk patients prior to a loss of receptor expression would provide benefit.

There is a larger body of research evaluating the role of PPARγ activation in melanoma. Nunez *et al*. evaluated the role of TZDs in an amino acid deprivation experiment and showed that TZD could promote apoptosis in A375 at 48 hours, albeit with very high doses of ciglitazone (50 μM)[22]. We have shown a significant increase in apoptosis in A375(DRO) at 6 days with only 1 μM PIO[7].

Weng *et al*. demonstrated that with modified TZD molecules that no longer bound and activated PPARγ, they could still induce antiproliferative and apoptotic effects on a variety of unrelated cancer cells[23] suggesting that TZDs had anticancer effects unrelated to PPARγ activation. However, these studies utilized high concentrations of compound (10 μM) to see any effect. In contrast, Aiello and colleagues demonstrated a complete blunting of the antiproliferative effect of TZDs on a variety of responsive anaplastic thyroid cancer (ATC) cells when PPARγ expression was diminished by siRNA and TZD concentrations of 10 μM were used for 4 days. A significant component of growth inhibition of cancer cells upon agonist activation of PPARγ is due to up-regulation of the cell cycle inhibitor p21^WAF1/CIP1 ^as demonstrated by Copland *et al*[24]. Using a PPARγ antagonist (GW9662) and siRNA of PPARγ in poorly differentiated cancer cells, the authors showed a blunting of promoter activity, antiproliferative activity and p21^WAF1/CIP1 ^up-regulation by PPARγ activation.

Only one other study has evaluated combination therapy with a rexinoid and TZD of a moderately differentiated carcinoma xenograft model. Cesario and colleagues [25] used a combination of 50 mg/kg/day LGD1069 and 15 mg/kg/day rosiglitazone and showed decreased tumor growth compared to either ligand alone. In our nude mouse model, we have previously shown that 100 mg/kg/day of LGD1069 markedly decreases A375(DRO) tumor growth and that this is associated with increased necrosis[8]. Using the same study design, the addition of 10 mg/kg/day of ROSI allows a similar tumor response with only 30 mg/kg/day of LGD1069. The *in vivo *effect of combination treatment seems to act primarily via decreasing growth as opposed to causing cell death as these tumors were the smallest and displayed the least amount of necrosis on a careful histological examination. These findings translate our previously published *in vitro *findings with A375(DRO) to an *in vivo *model[7].

The combination of retinoids and TZDs has been studied in two other models of cancer. Yamazaki [26]*et al*. transfected mutant (to mimic the unphosphorylated, active form) RXRα into Caco2 cells (that have endogenous expression of PPARγ) and then treated with relatively high doses of 9-cis RA (5 μM), which has both RXR and RAR agonist properties, and ciglitazone (10 μM). They found a synergistic decrease in viable cell counting and increase in apoptotic markers. Similarly, Hashimoto and colleagues [27] used a combination of 9-cis RA and troglitazone in the same concentrations on the KYSE series of esophageal carcinoma cell lines that all express high levels of RXRα and variable (though present) levels of PPARγ. They found a synergistic decrease in cell number, increased PARP cleavage and increased cell number in sub-G1 cell cycle phase with combination treatment.

Our study is unique in that we have evaluated the functional contribution of nuclear hormone receptor integrity to rexinoid and TZD activation of RXR and PPARγ receptors. We have demonstrated this functional partnership in A375(DRO) (likely a heterodimer) by two separate methods. The first strategy was a model of forced resistance to rexinoid and TZD therapies *in vitro*. The ability to create resistant cell lines to previously effective therapies has been demonstrated in previous studies including forced resistance to gemcitabine and paclitaxel in NSCLC [28,29]. These studies utilized a pulse therapy approach to generate drug resistance, whereas we have performed an increasing dose titration of LGD1069 and TZD. These experiments gave us insight into the relative importance of PPARγ in this model as RXRγ and RXRα levels were maintained, while PPARγ levels were decreased, even in the setting of resistance to LGD1069. This observation was supported by a direct shRNA experiment where knockdown of PPARγ markedly attenuated not only the antiproliferative response of TZD therapy, but also LGD1069 treatment. In the converse experiment, though affected, the response to TZD was not nearly as affected by RXRγ knockdown, though LGD1069 response was blunted. This suggests that in the A375(DRO) cell model, PPARγ plays a more prominent role in mediating the transcriptional response to either an RXR agonist, PPARγ agonist or the combination. The implication for clinical analysis of resistant tumors to nuclear hormone targeted therapies is that loss of expression of an "off-target" receptor may generate resistance if it is in a heterodimeric partnership with the "on-target" nuclear hormone receptor. Conversely, co-expression of RXR and PPARγ may suggest a higher potential response to either bexarotene or TZD alone, or the combination.

## Conclusion

In conclusion, combination therapy of a rexinoid with a retinoid or other nuclear hormone receptor, such as PPARγ, has the potential to decrease tumor growth in poorly differentiated cancers with the appropriate protein targets. Loss (or loss of function) of a nuclear hormone receptor that would otherwise act as a heterodimer partner may explain why targeted therapies to nuclear hormone receptors have limited or no efficacy. In general, as tumors become poorly differentiated and more widespread, they tend to lose expression of retinoid receptors[21,30,31]. Thus, earlier targeted therapy in aggressive variants of cancers may provide benefit by preventing aggressive biological behavior and dissemination.

## Methods

### Chemicals

All cancer cells were grown in RPMI 1640 media (Invitrogen Corporation, Carlsbad, CA) supplemented with 2% fetal bovine serum (FBS) (Hyclone, Logan, UT) and 0.2% Penicillin/Streptomycin. LGD1069 was provided by Ligand Pharmaceuticals (San Diego, CA). We utilized two TZDs; rosigllitazone (ROSI) was provided by GlaxoSmithKline (Barnard Castle Durham, UK) and pioglitazone (PIO) was provided by Takeda Pharmaceuticals (Osaka, Japan).

### Cell Lines

DRO90-1 was provided by Dr. G.J. Juillard (UCLA). BHP 5–16 was provided by Dr. Jerome Hershman (UCLA). These cancers have been shown to be misidentified as follows: DRO90-1 matches the STR profile of A375 (melanoma), BHP 5–16 matches M14 (melanoma) [15]. Thus, by our own convention, these cell lines will be named as follows: A375(DRO) and M14(5–16). This name identifies the parent cell line, but distinguishes them as unique sub-lines that may have differential responses to similar treatments for A375 or M14 in another lab.

### Xenograft Model

Athymic nude mice were purchased from National Cancer Institute (NCI – NCr-*nu/nu *01B74). All mice were male, 6–7 weeks old weighing 15–30 grams. Mice were handled in accord with the approval of the UCDHSC Animal Care and Use Committee. The groups were defined as: control chow, LGD1069 (30 mg/kg/day), ROSI (10 mg/kg/day) and LGD1069/ROSI (30/10 mg/kg/day respectively).

A375(DRO) and M14(5–16) cells were grown in RPMI media supplemented with 2% FBS and suspended at 5 × 10^6 ^cells/200 μL sterile PBS. Mice were separated into groups of 8 and after they were anesthetized with an intraperitoneal injection of Avertin (0.5–0.7 cc of 32 mg/mL), 5 × 10^6 ^tumor cells were injected subcutaneously on the R. flank of each mouse.

LGD1069, ROSI or the combination was blended into LabDiet 5001 by TestDiet – a division of Purina Mills, at a dose estimated to deliver the desired dose based on the assumption that mice would weigh 20 grams and eat 5 grams of chow/day (based on previous experience). The LabDiet 5001 alone was used as control chow. Each diet was irradiated with Cobalt 60 gamma irradiation to sterilize the chow for nude mouse consumption. Irradiation of LGD1069 powder did not affect the ability to inhibit cancer cell growth *in vitro *[8]. Mice were weighed prior to tumor injection, and food was weighed every two weeks to estimate the amount of consumption per mouse. Treatment chow was started after tumors reached a volume of ~100–200 mm^3^.

### Tumor Assessment

Mice were observed twice per week and tumors were measured with electronic calipers. Tumor volume was estimated using the formula: tumor (length × width × height)/0.5236. Based on previous experience and our animal care facility requirements, the study was designed to stop when the first group of 8 mice had an average tumor size of 3000 mm^3 ^for A375(DRO). The M14(5–16) cell line demonstrated slower growth, thus the tumors were allowed to grow for 6 weeks. These time points were chosen to maximize the differences between groups yet prevent significant morbidity as it had been observed that mice with tumors at these time points were still mobile, able to access food and water easily, and had not lost significant weight.

Tumor necrosis was assessed by a pathologist blinded to treatment conditions. The percent of tumor necrosis per tumor was scored and averaged to allow for statistical comparison between groups (1-way ANOVA – SigmaStat).

### Ligand Resistant Cell Lines

In order to determine the relative importance of RXR and PPARγ receptors in A375(DRO), we used a novel approach of developing sub-lines resistant to previously effective ligands by passaging the cells in media with slowly increasing drug concentration. We began the titration with LGD1069, PIO and the combination (1/2 of each ligand) at the ineffective dose of 10 nM and increased the concentration every 1–2 weeks until the cells were growing in the previously inhibitory concentration of 100 nM. A control population of A375(DRO) were grown in volume equivalent vehicle (DMSO) as a control for increasing passage number. The approximate number weeks for dose titration for all treatment conditions was 14 weeks (+/- 1 week) until cells were growing at a similar rate in 100 nM of all 3 treatment conditions or volume equivalent vehicle. Cell identification for these newly resistant sub-lines is designated by the name of the exposed ligand followed by "R" for resistant (LGD1069 R; TZD R; LG/TZD R). The matched cells passed in media with volume equivalent vehicle are designated DMSO R.

### shRNA

We used a lentiviral mediated shRNA system from Sigma (St. Louis, MO) and followed the manufacturer's protocol. Lentiviral particles contain shRNA toward RXRγ or PPARγ-specific sequences as well as a scrambled (SCR) sequence that consists of 5 nucleotides that do not match any known gene transcript in both the murine and human genome. The infected cells are selected by a puromycin resistance and then assessed for correct insertion/RNA inhibition by qRT-PCR or western blot for either RXRγ or PPARγ. The concentration of puromycin used to select for DNA construct incorporation cells was 0.4 μg/mL.

### Quantitative Reverse transcription-PCR (qRT-PCR)

Total RNA was isolated from A375(DRO) shPPARγ using the RNeasy Mini Kit (Quiagen, Valencia, CA) as per the manufacturer's protocol. The mRNA for PPARγ was measured by real-time Quantitative RT-PCR using ABI PRISM 7700. The sequences of forward and reverse primers as designed by Primer Express (PE ABI) were 5'-AGT GGA GAC CGC CCA GGT-3' and 5'-GGG CTT GTA GCA GGT TGT CTT G-3'.

The TaqMan™ fluorogenic probe used was 6FAM-TGC TGA ATG TGA AGC CCA TTG AAG ACA-TAMRA.

Amplification reactions, thermal cycling conditions and generation of a standard curve have been described previously[8].

### Western Blot Analysis

Nuclear extracts were obtained from all four resistant A375(DRO) sub-lines, A375(DRO) shPPARγ, A375(DRO) shRXRγ and M14(5–16) shRXRγ for analysis of RXRα, RXRγ, RARβ (M14(5–16) only) and PPARγ (A375(DRO) only) proteins utilizing a nuclear extract kit from Active Motif (catalog #400100, Carlsbad, CA). The protein content of lysates was measured utilizing a commercial protein assay kit – DC from Bio-Rad (Hercules, CA). 60 μg of protein was loaded for each sample and the gel and blot were carried out as previously described [7]. RXRγ (MS-1343-P NeoMarkers) and RXRα (sc D-20) antibodies were used at a concentration of 1:500 and PPARγ (H-100) rabbit polyclonal ab (sc-7196, Santa Cruz Biotechnology, Santa Cruz, CA) was used at 1:500. RARβ (sc-552, Santa Cruz Biotechnology, Santa Cruz, CA) was used at a dilution of 1:500. After washing, membranes were incubated for one hour at room temperature with anti-rabbit IgG conjugated to horse-radish peroxidase at a 1:5000 dilution for RXRs, 1:1000 for PPARγ and 1: 5000 for RARβ (GE Healthcare UK). β-actin or Poly (ADP-ribose) polymerase (PARP) protein was probed for loading control. The ECL detection reagent from Amersham Biosciences (Piscataway, NJ) was used for immunodetection.

### Cell growth and proliferation

All four resistant A375(DRO) cell lines, A375(DRO) shPPARγ, A375(DRO) shRXRγ and M14(5–16) shRXRγ with proper SCR control conditions were grown to approximately 80% confluence in 100 mm tissue culture plates. Cells were then harvested using Trypsin-EDTA (Invitrogen Corporation, Carlsbad, CA) and counted using a hemocytometer. Cells were then transferred to a 96-well plate at a concentration of 500 cells/200 μl of media. Each row of eight wells received the same cell type and subsequently the same drug. After cells were allowed to plate down overnight, media was aspirated and media with the appropriate concentration of ligand or equivalent volume of vehicle was added to each well. Fresh media with vehicle or ligand was added every 72 hours. At the completion of 6 or 9 days (depending on the experiment), cell proliferation was assessed following the manufacturers instructions using the CellTiter 96 Aqueous Non-Radioactive Cell Proliferation Assay (Promega, Madison, WI). Following a two-hour incubation at 37°C, each plate was analyzed by a MRX Micro plate Reader (Dynatech Laboratories, Chantilly, VA) using Revelation software.

## Competing interests

The authors declare that they have no competing interests.

## Authors' contributions

JPK conceived of the study, performed the animal experiments and resistant cell line experiments and helped draft the manuscript; VS performed the shRNA experiments and western blot analyses; AB performed the resistant cell line experiments and animal experiments; WRH performed the animal experiments; ML performed the shRNA experiments; UP performed the qRT-PCR experiments; SS analyzed and reported the histological review of tumor xenografts; BRH conceived of the study and helped draft the manuscript.
